# The differential effects of dynamic, static, and combined activities in forest bathing on health outcomes by gender in older adults: evidence from a national forest park trial

**DOI:** 10.3389/fpsyg.2025.1648144

**Published:** 2025-10-22

**Authors:** Menglei Yin, Zhiman Xu, Xiaojun Zheng, Rui Jiao, Kankan Li

**Affiliations:** 1 College of Landscape Architecture and Art, Northwest Agriculture and Forestry University, Xianyang, China; 2 College of Horticulture, Nanjing Agricultural University, Nanjing, China

**Keywords:** forest bathing, national park, natural therapy, dynamic and static activities, gender differences, older adult health, mental well-being

## Abstract

**Background:**

Forest bathing, as a form of natural therapy, has been increasingly recognized for its therapeutic effects on physiological and psychological health. National parks, as protected natural environments, provide ideal settings for such interventions, yet empirical studies conducted directly within these landscapes remain limited.

**Objective:**

This study aims to compare the effects of dynamic, static, and combined dynamic-static activities on the health outcomes of older adult populations of different genders, within a real national park environment.

**Methods:**

Seventy-two middle-aged and older adults (mean age 62.5 ± 7.22 years) were divided into four groups: combined dynamic-static, dynamic, static, and control. Physiological (EEG, HR, skin conductance, SBP, DBP) and psychological indicators (BPOMS, PRS) were measured.

**Results:**

The combined dynamic-static group showed the best overall improvements, particularly in positive emotions and blood pressure. The dynamic group excelled in diastolic pressure and perceived restoration, while the static group improved vitality and reduced fatigue. Males showed more physiological improvements, whereas females excelled in psychological restoration across all activities.

**Significance:**

Conducted in the ecologically rich Panda Valley of Shaanxi Province -a core area of the Giant Panda National Park-this study provides real- world evidence that national parks serve as effective therapeutic landscapes. It offers scientific justification for integrating nature-based therapies into public health strategies and enhancing the health value of national parks.

**Conclusion:**

Forest bathing activities within national parks can serve as a powerful natural therapy for promoting older adult health, tailored by activity type and gender.

## 1 Introduction

### 1.1 Health challenges and gender differences in an aging population

With an aging population, health issues among the middle-aged and older adult are increasingly concerning. They often face cardiovascular diseases, hypertension, diabetes, dizziness, anxiety, loneliness, functional disorders, and high incidences of chronic diseases such as depression (Ambelu and Teferi, 2023; Ma et al., 2018; Wen et al., 2023). These mental health issues may stem from the complex process of identity transitions, changes in social roles, and lifestyle adjustments (Shafiq et al., 2023). Chronic diseases do not only affect the quality of life of the older adult but also increase medical burdens and societal costs (Hung, 2024). In China, hypertension remains highly prevalent. A recent survey in Tianjin reported a prevalence of 46.8% among adults. Regarding mental health, a systematic review and meta-analysis estimated that 20.0% (95% CI: 17.5–22.8%) of Chinese older adults experience depressive symptoms, compared with a global prevalence of 28.4% (95% CI: 24.8–32.0%) (Wang et al., 2025). These conditions can be managed with interventions and preventive measures, improving the older adult’s health, delaying aging, and enhancing quality of life (Singh et al., 2023). Studies have shown that physical activities and psychological empowerment can prevent and manage post-retirement mental health issues in the older adult (Shafiq et al., 2023). However, significant gender differences exist in disease prevalence among middle-aged and older adult groups. Women show higher depression symptoms with chronic diseases, while men are more prone to cardiovascular diseases from smoking and drinking. In certain regions, women show rising hypertension prevalence due to various factors (Weisberg et al., 2011; Xu et al., 2016).

### 1.2 Comprehensive health benefits of forest bathing for middle-aged and older adult people

Forest bathing offers effective prevention and management for this demographic. Originally proposed in Japan in the 1980s, forest bathing (FB, Shinrin-yoku) refers to immersing oneself in a forest environment through multiple senses, rather than engaging in a single prescribed activity (Li, 2010). It is a primary forest therapy. Forest environmental factors like Phytoncides (Thangaleela et al., 2022), natural sounds, scenery, and negative ions improve mood, promote mental health, enhance immunity, alleviate pain and stress, and have anti-cancer effects (Weisberg et al., 2011; Li et al., 2008; Ulrich, 1984). Additionally, forest bathing positively impacts both physiological and psychological health. Studies have shown that forest walking not only benefits physical health but also significantly reduces stress. It boosts positive emotions, reduces negative ones, lowers cortisol and blood pressure, enhances parasympathetic activity, reduces norepinephrine and dopamine, and increases anti-stress hormones (Chen et al., 2025). Horiuchi et al. noted forest walking is especially beneficial for the older adult, lowering blood pressure and improving emotional states (Horiuchi et al., 2015; Li et al., 2011; Song et al., 2019). Research indicates that the benefits of forest bathing on mental and physical health are more pronounced in more natural and pristine forest environments (Chen et al., 2019). Unlike urban botanical gardens or small forest patches, national forest parks offer richer biodiversity and fewer human disturbances, crucial for assessing forest bathing’s true effects. Thus, our study was conducted in an authentic national forest park.

### 1.3 Different types of forest bathing activities and their health benefits

Most forest bathing research focuses on immune boosting and stress reduction. However, these studies often treat forest bathing as a unified activity, without distinguishing the physiological and psychological effects of different activity modes. In reality, forest bathing encompasses various activities, including dynamic movements like hiking and static practices like meditation. The health benefits of different activity types, as well as their combined effects, remain underexplored. In this study, we do not redefine forest bathing as specific activities; instead, we regard dynamic, static, and combined modes as different ways of engaging in the broader practice of spending time in forests. This study categorizes forest bathing into dynamic, static, and combined modes.

Dynamic forest bathing involves continuous physical activities like hiking, running, and cycling. We selected forest hiking for our study. Exercise promotes blood vessel elasticity and function, increases blood flow, and reduces blood pressure and vascular resistance, crucial for hypertensive middle-aged and older adult individuals (Edwards et al., 2023). Additionally, exercise increases the high-frequency component of heart rate variability (ln HF) and decreases the low-frequency/high-frequency ratio (ln LF/ln HF), indicating enhanced parasympathetic and reduced sympathetic activity (Ambelu and Teferi, 2023). For mental health, exercise promotes the release of endorphins and serotonin, alleviating negative emotions, enhancing mood, and improving self-efficacy (Lenasi and Šijanec, 2023; Lim et al., 2008). It increases cerebral blood flow, oxygen supply, promotes neuronal plasticity, new connections, and enhances cognitive function and memory (Xu et al., 2024).

Static forest bathing involves stationary activities like meditation, sitting, and deep breathing, focusing on isometric muscle contraction and posture maintenance. We selected forest meditation for our study (Li et al., 2008; Wen et al., 2019). Meditation indirectly promotes NK cell activity, enhancing immunity. It regulates the autonomic nervous system, increasing parasympathetic and reducing sympathetic activity, helping the body cope with stress. Furthermore, meditation enhances brain plasticity, particularly in the prefrontal cortex and insular regions related to emotion regulation and self-awareness, thereby improving mental health (Ambelu and Teferi, 2023).

The combined dynamic-static forest bathing mode integrates both activity types to enhance health benefits, reflecting real-world forest therapy practices that include both physical activity (e.g., hiking) and relaxation (e.g., meditation). However, no studies have systematically compared its effects with dynamic or static activities alone. This study addresses this gap, providing scientific insights to optimize forest therapy programs.

### 1.4 Gender differences and responses in forest bathing activities

During forest bathing activities, men and women exhibit different responses due to their physiological and psychological structural differences (Fernandez-Pena et al., 2023; Kelly et al., 2008; Viertio et al., 2021; Wang et al., 2007). Gender factors have always been a focus of researchers. Men have higher heat tolerance, which is related to their body temperature regulation mechanisms and sweat gland function (Fernandez-Pena et al., 2023). Men have lower sweat gland density but higher sweating efficiency, allowing effective heat dissipation and maintenance of core temperature (Barba et al., 2008). In high-temperature or sun-exposed forest areas, men adapt better; however, they may be less comfortable in cold environments (Foll et al., 2013). Additionally, men generally have higher blood pressure and lower average resting heart rates, which are associated with cardiac output and myocardial contractility (Shafiq et al., 2023). Women, on the other hand, are more comfortable in cold environments, likely due to higher subcutaneous fat content, which provides better insulation (Barba et al., 2008). Women adapt better to cold environments and restore temperature balance faster, making them more suited to low-temperature forest areas (Weatherald et al., 2021). Women’s blood pressure is lower between puberty and menopause but rises after menopause due to decreased estrogen levels (Barba et al., 2008; Weatherald et al., 2021). Women have higher resting heart rates, stronger parasympathetic responses, and greater heart rate variability (HRV), indicating better autonomic cardiac regulation (Shafiq et al., 2023).

Men often use more coping strategies for negative emotions and experience less distress, possibly due to their larger amygdala, crucial for emotional processing (Weisberg et al., 2011). During forest bathing, men may achieve greater relaxation through dynamic activities (e.g., hiking), while women attain emotional release and balance through static activities (e.g., meditation). Women’s superior interhemispheric brain coordination explains their multitasking and emotional processing advantages (Maji, 2018). Our study will use EEG, skin conductance, heart rate, and blood pressure to explore gender differences in forest bathing, aiming for a precise gender analysis.

### 1.5 Research objectives and hypotheses

This study will examine how dynamic, static, and combined dynamic-static forest bathing affect physiological and psychological health in middle-aged and older adult individuals in a national forest park. It will: compare the impact on physiological indicators (EEG, HR, GSR, SBP, DBP); assess effects on emotional states (BPOMS) and environmental perception recovery (PRS); and analyze gender differences in responses, aiming to inform personalized health programs.

This research aims to address gaps in current studies and offer comprehensive guidance for promoting health in middle-aged and older adult individuals. It will also aid in designing effective forest bathing activities to maximize physical and mental health benefits.

This study explores whether combined dynamic-static forest bathing integrates the benefits of each activity and examines gender differences in various forest bathing activities. Thus, the following hypotheses are proposed:

1) Combined dynamic-static forest bathing may provide greater improvements in physiological and psychological health in middle-aged and older adult people compared with dynamic or static activities alone, though effects may vary across indicators.
2) There may be exploratory gender differences in the physiological and psychological impacts of dynamic, and combined dynamic-static forest bathing between middle-aged and older adult men and women. Each form of forest bathing may vary in its effects on reducing negative emotions, increasing positive emotions, and improving physiological indicators.

## 2 Research methodology

### 2.1 Materials and methods

#### 2.1.1 Study site

**Figure 1:**
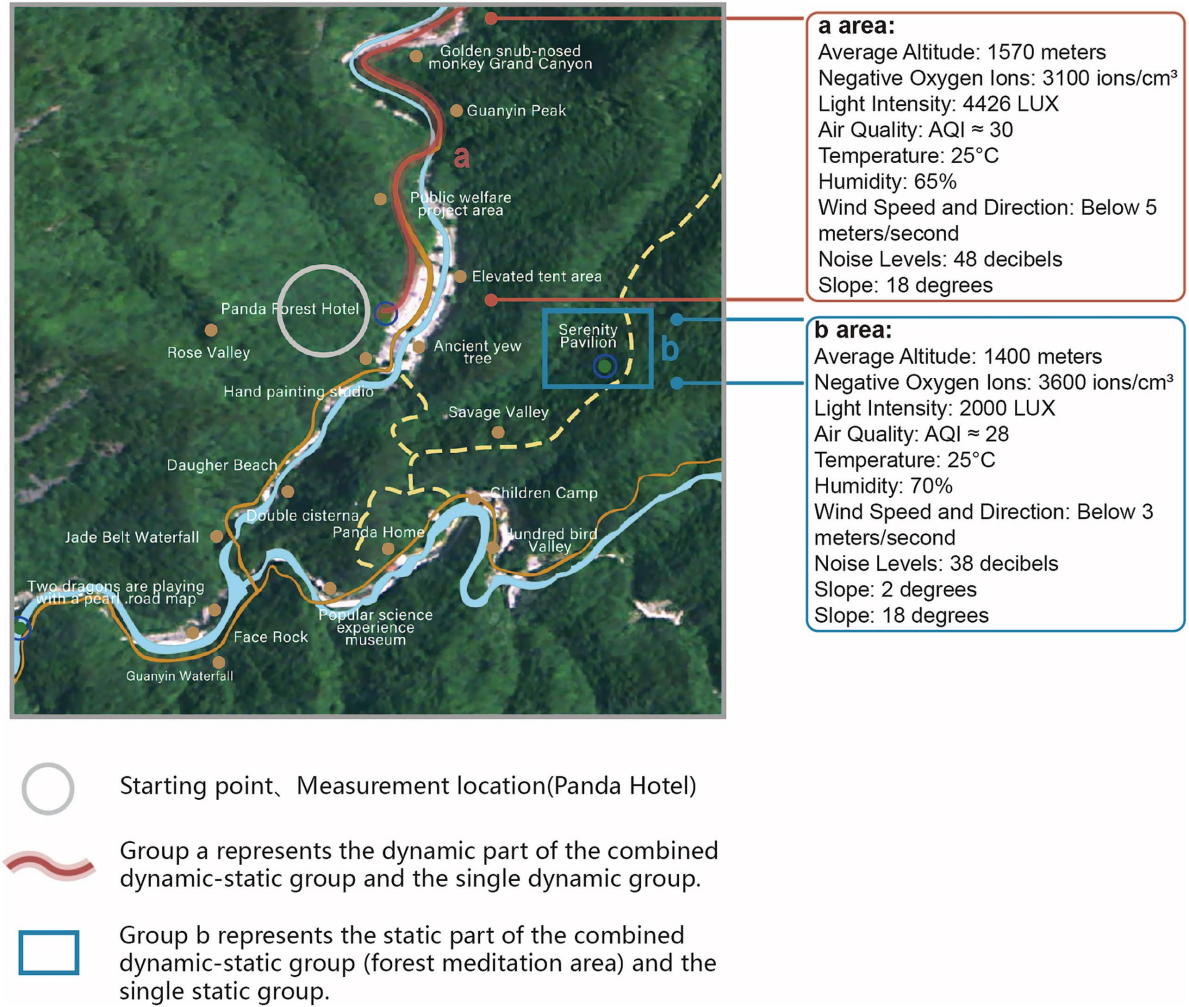
Experimental route map.

The experiment took place in April 2024 at Panda Valley National Forest Park, Foping County, Shaanxi (33.658°N, 107.807°E, elevation 1,080–2094 meters; see Figure 1). The park’s warm, humid climate and high forest coverage, with 25,000 negative ions/cm^3^, earned it the name “Forest Oxygen Bar.”

We divided participants into four groups: Group A performed combined dynamic-static forest bathing (60 min total: 30 min dynamic, 30 min static, with a 15 min break), Group B did dynamic forest bathing alone (30 min), Group C did static forest bathing alone (30 min), and Group D was a control group (30 min). The 60 min duration for the combined intervention was chosen to reflect common forest therapy practices, which typically last 45–60 min or longer and often integrate both dynamic and static phases (Park et al., 2010; Vermeesch et al., 2024; Zhang et al., 2023).

The intervention groups were tested in Panda Valley National Forest Park, Foping County, Shaanxi, due to its uniform open forest structure, minimizing coverage and spatial structure effects. The control group experiment was conducted in the Northwest A&F University College of Landscape Architecture and Arts laboratory. An environmental control system adjusted the lab’s temperature and humidity to match Panda Valley, ensuring experimental consistency and validity. This approach maintained comparable climatic conditions between the control and forest groups, minimizing confounding effects of temperature and humidity, while providing a non-natural baseline against which the effects of forest bathing could be highlighted.

To ensure scientific rigor, Groups A, B, and C used the same forest area with environmental controls. Experiments were scheduled at 9 a.m. to minimize light and temperature changes. Walking routes and sitting locations were consistent. Ecologists assessed plant species, air quality, noise, terrain safety, and wildlife absence. In the static group, participants engaged in guided forest meditation while wearing standardized headphones with a professionally developed meditation script. The script was carefully evaluated and validated by landscape architecture experts to ensure consistency and effectiveness (validation report available in Forest Meditation Evaluation Report and Text Version). This setup ensured that all participants received identical auditory input, minimizing variability and external environmental sound exposure.

Group A used Route 1, combining a walk from Panda Forest Hotel to Golden Monkey Grand Canyon and static activities at Jingxin Pavilion. Jingxin Pavilion, near the hotel, served as the starting point for all routes. Group B used only the walking route of Route 1, and Group C used only Jingxin Pavilion.

Group D’s experiment was conducted in the 12 m^2^ landscape lab at Northwest A&F University, isolated by PVC whiteboards to eliminate visual distractions. The experiment occurred from 9 to 11 a.m. in April, maintaining an indoor temperature of 21.5 °C and 60–80% humidity, with no external noise (Figure 1) (Wen et al., 2023; Mao et al., 2017). As Group D served as the blank control group to provide a baseline for comparison, no elements simulating the forest environment were introduced.

#### 2.1.2 Study participants

We recruited middle-aged tourist volunteers through online and offline channels in Foping County and Yangling District, Shaanxi Province, from early March to the end of April 2024. The trial was conducted in April from 9:00 to 11:00 a.m., with all participants completing the assigned tasks as planned. A total of 72 participants were enrolled (mean age 62.5 ± 7.22 years; range 60–75), with a gender ratio of 1:1. To ensure homogeneity and reliability, individuals who were smokers, drinkers, menstruating, experiencing physical discomfort, or with olfactory/hearing impairments or severe health conditions (e.g., cancer, heart disease, mental illness) were excluded (Yau and Loke, 2020).

All participants provided informed consent and were free to withdraw at any time, though no withdrawals or exclusions occurred during the study. Each volunteer was assigned a unique ID number and received a small gift after participation. The study protocol adhered to the ethical standards of the Psychology Center at Northwest A&F University and the Declaration of Helsinki. It was approved by the Psychology Development and Education Center of Northwest A&F University (Approval No. 202403121185) and registered under NWAFU202407643.

**Figure 2:**
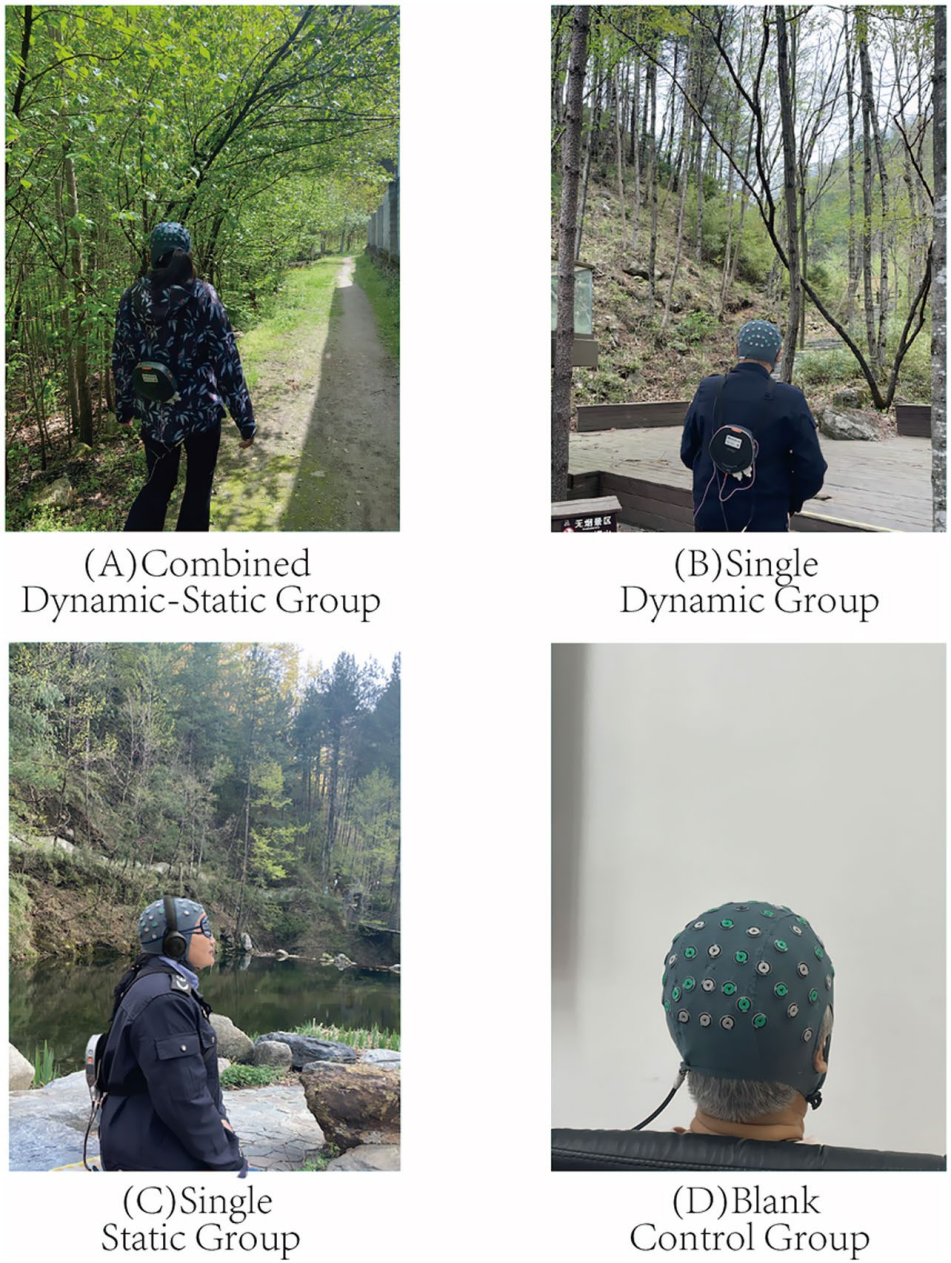
Experimental grouping: **(A)** Combined Dynamic-Static Group, **(B)** Single Dynamic Group, **(C)** Single Static Group, **(D)** Blank Control Group.

To minimize the Hawthorne effect, participants were not informed of the study objectives. Participants were randomly assigned to one of four groups (A–D) through a computer-generated sequence by an independent researcher, with allocation concealed in sequentially numbered opaque envelopes; both participants and investigators remained blinded until the intervention began. Each group consisted of 18 participants (9 males and 9 females), totaling 72. All participants completed their assigned activities and were included in the primary outcome analysis (Figure 2). This sample size is methodologically robust and consistent with previous forest bathing studies, which typically enrolled 12–20 participants per group. However, as each gender subgroup contained only nine participants, the statistical power for subgroup analyses was limited.

### 2.2 Study metrics

**Figure 3:**
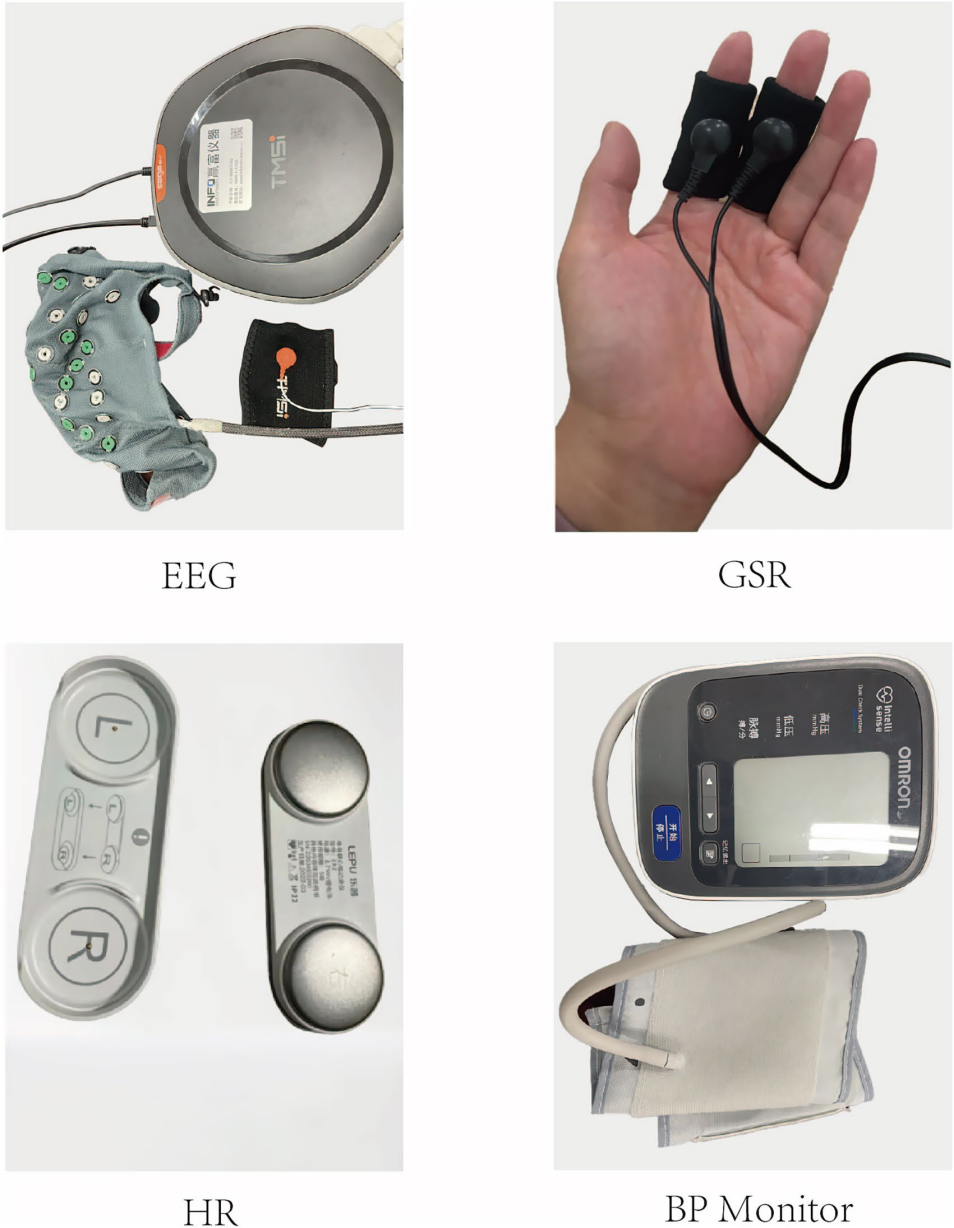
Experimental equipment.

This study determined physiological and psychological indicators to quantify the effects of dynamic and static forest bathing activities on participants’ recovery, based on the experimental design, real forest environment, and available equipment. The physiological indicators included the portable EEG device SAGA 64 + (with electrodes placed according to the international 10–20 system) from the Dutch company TMSI, portable skin conductance (Arduino OLED), heart rate (Lepu ECG recorder, ER2 version), and Omron blood pressure monitor (HEM-7124). Psychological indicators were measured using the Perceived Restorativeness Scale (PRS, 5-point scale) and the Brief Profile of Mood States (BPOMS, 5-point scale) (Figure 3). These indicators were selected based on their established use in research on stress reduction, autonomic nervous system regulation, and emotional well-being. Previous studies have shown that the benefits of physical activity for older adult health are demonstrated through both physiological and psychological factors. Blood pressure, heart rate, skin conductance, and EEG were chosen as indicators of the physiological state, with EEG specifically reflecting relaxation and pleasure levels. The PRS and BPOMS self-assessment scales were used to assess participants’ psychological state.

#### 2.2.1 Psychological indicators

The Perceived Restorativeness Scale (PRS) assesses psychological restoration in various environments. Participants rate descriptive items on a five-point scale from 1 (strongly disagree) to 5 (strongly agree). Higher scores indicate higher perceived restoration. PRS is based on Attention Restoration Theory (ART), which suggests that exposure to natural environments helps reduce cognitive fatigue and enhances well-being (Liu et al., 2024). Since forest bathing is designed to promote relaxation and mental recovery, PRS is an appropriate tool for measuring its psychological benefits (Takayama et al., 2022). This study uses PRS to measure perceived restoration under different activities (Wen et al., 2023; Mao et al., 2017).

The Brief Profile of Mood States (BPOMS) assesses mood, covering dimensions like pleasantness, tension, and fatigue. Participants rate each item from 1 to 5. This study uses BPOMS to evaluate mood changes under different activities. The questionnaire is in Chinese, with explanations provided. Data are used for research and education, ensuring privacy (Maji, 2018; Mao et al., 2017). BPOMS was chosen for its conciseness and efficiency, especially for our older adult participants, who may find lengthy questionnaires challenging. This simplified version has been widely validated in both recent and past studies, demonstrating high reliability and strong correlation with the full POMS, making it a justified and effective tool for our research (Cella et al., 1987; Shao et al., 2024; Shacham, 1983; Yan et al., 2024; Yoo et al., 2005; Zhao et al., 2024).

#### 2.2.2 Physiological indicators

Electroencephalography (EEG) measures brain responses to stimuli and is a key physiological indicator. We used the portable SAGA 64 + EEG from TMSI, adhering to the 10–20 system for electrode placement. This device provides high-quality data, filtering out noise and muscle interference, and is suitable for field and lab research. We focused on frontal electrodes (Fp1, Fp2, F3, F4, F7, F8) because frontal EEG activity is strongly associated with emotional and cognitive states. Specifically, Left frontal electrodes (Fp1, F7, F3) correlate with positive emotions (Zhao et al., 2018), while right frontal electrodes (Fp2, F8, F4) correlate with negative emotions). We used TMSI’s algorithms to analyze EEG data from these electrodes, obtaining alpha wave data per second in the frontal area. Increased alpha wave activity and decreased beta wave activity have been linked to reduced stress and improved mental clarity (Grassini et al., 2022).

Galvanic Skin Response (GSR) measures skin conductivity, reflecting emotional responses, stress, and autonomic nervous system activity. GSR is particularly relevant for assessing emotional arousal in response to environmental stimuli, such as the calming effects of natural settings. Increased skin conductance indicates emotional tension and sympathetic activation, while decreased conductance signals calmness (Kim et al., 2019). GSR has been widely applied in stress and environment-related studies.

Heart Rate (HR) indicates cardiac activity and cardiovascular function. Variability in HR reflects health, emotional state, and response to stimuli. Increased HR can result from excitement, activity, or stress, while decreased HR occurs with relaxation. HR is particularly useful in forest therapy research as it reflects both acute physiological relaxation and long-term cardiovascular benefits. Previous studies have confirmed its sensitivity to autonomic regulation and stress reduction.

Blood Pressure (BP) is the force of blood on vessel walls, comprising Systolic (SBP) and Diastolic Pressure (DBP). BP is affected by cardiovascular status, vessel elasticity, blood viscosity, and autonomic regulation. Stress, tension, or activity raises BP, while relaxation lowers it. Since forest bathing has been linked to reductions in stress-related hypertension and improved cardiovascular health, BP serves as a critical measure of the physiological benefits of exposure to natural environments (Yau and Loke, 2020; Ideno et al., 2017; Peterfalvi et al., 2021).

### 2.3 Experimental procedure

#### 2.3.1 Combined dynamic-static forest bathing group (Group A)

This study designed a combined dynamic-static forest bathing group (Group A) to explore its effects on physiological and psychological health and compare different methods. Volunteers were randomly assigned and transported by shuttle bus at 8 a.m., given 30 min to rest and acclimate.

**Figure 4:**
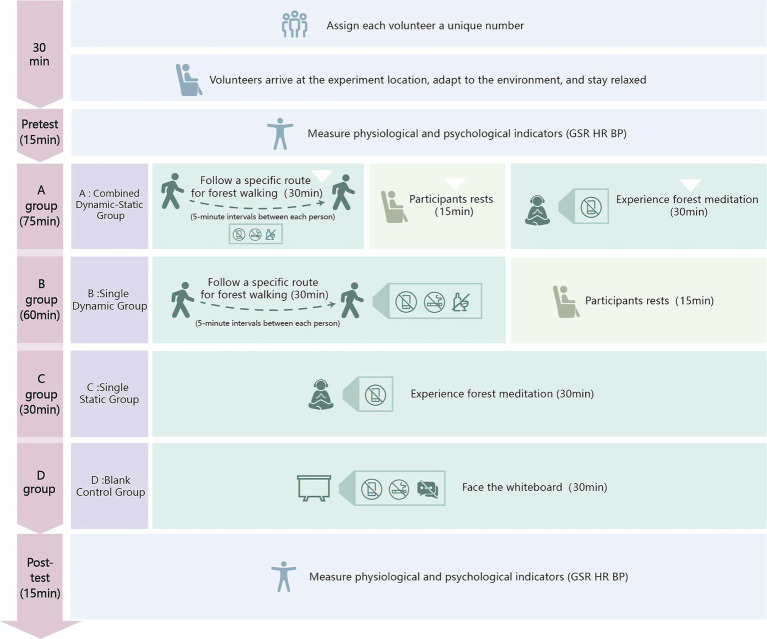
Experimental procedure.

Participants began with dynamic forest hiking. Electronic devices and alcoholic or caffeinated beverages were prohibited. Hikes started at 5 min intervals, followed by a 15 min rest. Next, they moved to the static phase, receiving cushions, eye masks, and headphones for 30 min of forest meditation. Physiological and psychological indicators were measured pre- and post-experiment. Participants received small gifts afterward (Figure 4).

#### 2.3.2 Dynamic forest bathing group (Group B)

The dynamic forest bathing group (Dynamic Group) was formed by random assignment. Volunteers were given unique IDs, and their health, drinking, and smoking habits were confirmed. The procedure was described objectively to prevent psychological suggestion, such as creating expectations about relaxation, mood improvement, or health benefits.

The schedule was as follows: Volunteers arrived at 8 a.m., rested for 30 min, and had baseline data measured. The experiment started at 9 a.m. The dynamic group hiked a 3-km route at 5 min intervals, with a target duration of 30 min (acceptable range: 28–33 min) along a trail ranging from 1,400 to 1,570 meters in altitude. To help maintain a consistent walking pace (~6 km/h), research staff provided a brief pacing demonstration before departure, and visual time markers were placed along the route for self-regulation. However, volunteers were instructed to walk at a comfortable, natural speed, and only those who completed the walk within the 28–33 min range were included in the final analysis. This hiking route was located within Foping Panda Valley National Forest Park, Shaanxi Province, where the overall elevation spans from 1,080 to 2094 meters. Electronic devices, alcohol, and smoking were prohibited. After the hike, volunteers rested for 15 min before their physiological and psychological indicators were remeasured. Gifts were distributed only after all measurements had been completed to ensure they did not influence volunteers’ responses.

#### 2.3.3 Static forest bathing group (Group C)

The static forest bathing group (Static Group) was formed by randomly selecting volunteers, each with a unique ID. The activity began at 8 a.m., with volunteers shuttled to the site. After 30 min of acclimation, baseline measurements were taken for EEG, heart rate, blood pressure, and psychological stress.

Participants were provided with cushions, disposable eye masks, and JBL TUNE 500BT headphones. The forest meditation instructions, validated by experts in landscape architecture, were played at a baseline volume of 50 decibels. Before the experiment, participants were asked to confirm whether they could clearly hear the audio and were allowed to adjust the volume slightly within a comfortable range (typically 50–60 dB) to suit individual preferences (Moore, 2012). The 30 min session began at 9 a.m. Mobile phones and removal of eye masks or headphones were prohibited. Participants could signal to end the experiment if uncomfortable. Post-meditation, physiological and psychological indicators were measured to assess the static forest’s health impacts. Participants received small gifts. This study explored the benefits of static forest environments on health.

#### 2.3.4 Blank control group (Group D)

The blank control group (Group D) provided a baseline for assessing environmental impacts on participants’ psychological and physiological responses. The experiment was conducted in the College of Landscape Architecture’s laboratory at Northwest A&F University, with standardized conditions to minimize interference. White PVC boards divided the lab into individual units to limit participant interaction.

The control group session lasted 30 min. Participants were prohibited from talking, smoking, or using devices. Heart rate, blood pressure, mood, and perceived restorativeness were measured pre- and post-experiment to evaluate environmental impact and establish a baseline. Gifts were given at the end to encourage participation.

### 2.4 Data processing

In this study, data processing was initially performed using Microsoft Excel 2019 for statistical organization and arrangement. Subsequently, SPSSAU software was used for data analysis (Mingming et al., 2024; Li T., 2023). This randomized controlled trial divided participants into four groups: Group A (combined dynamic-static), Group B (dynamic), Group C (static), and Group D (control). Data were presented as mean ± standard deviation, with normality tests performed.

Non-normally distributed data were analyzed with the Wilcoxon signed-rank test. For normally distributed data, paired *t*-tests were conducted, and when multiple comparisons were involved, Holm step-down correction was applied to control the family-wise error rate (*p* < 0.05). Effect sizes were reported as Cohen’s *d* with 95% confidence intervals (Song et al., 2019; Song et al., 2017). Sex was also included as a covariate in the statistical models to test main and interaction effects, thereby enhancing the robustness of the results. To reduce potential bias caused by different intervention durations, relative change values (*Δ* = post – pre) were calculated and used as the primary outcomes, ensuring that results reflected intervention type rather than exposure time. Data visualization utilized Photoshop 2019 and Origin 2021.

## 3 Research results

This study used a pre-test and post-test design to compare effects of various forest bathing activities on physiological and psychological indicators. Groups included were A (combined dynamic-static), B (dynamic), and C (static). The goal was to measure changes in physiological (EEG, GSR, heart rate, SBP, DBP) and psychological (BPOMS TMD, PRS) indicators before and after the activities.

Baseline measurements showed no significant differences (*p* > 0.05) (Supplementary Table 1). The study aimed to explore how forest exposure with varying activity intensities affects participants’ physiological and psychological health, including gender differences.

### 3.1 Comparison of physiological effects of different activity types on middle-aged and older adult groups

#### 3.1.1 Electroencephalography

**Figure 5:**
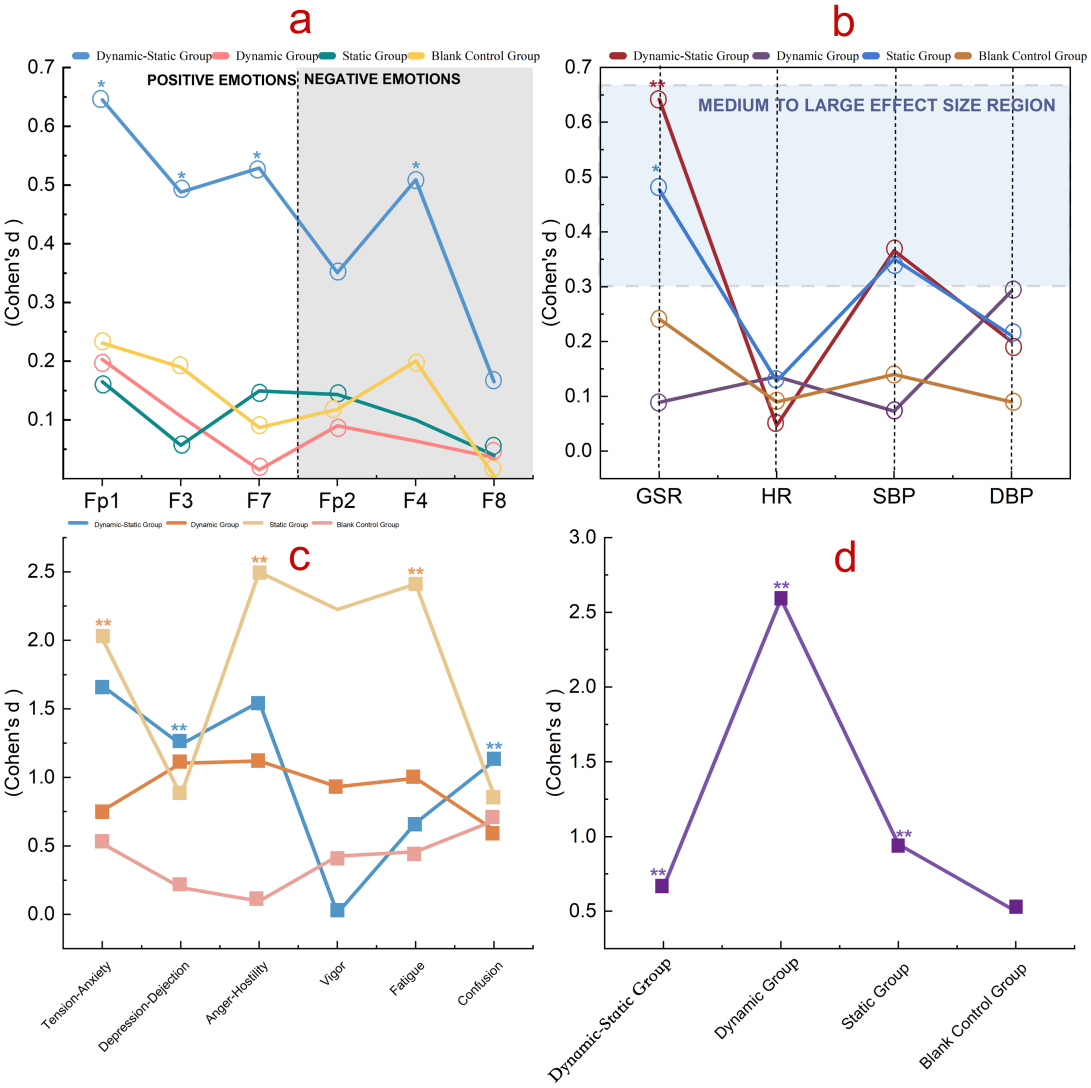
Differences in physiological and psychological indicators across groups. **(a)** EEG effect sizes at Fp1, F3, F7 (positive-emotion channels) and Fp2, F4, F8 (negative-emotion channels). **(b)** Effect sizes for GSR, HR, SBP, and DBP. **(c)** BPOMS subscales: tension–anxiety, depression–dejection, anger–hostility, vigor, fatigue, confusion. **(d)** PRS total by group (Dynamic–Static, Dynamic, Static, Blank Control). Lines and symbols denote the four groups. Values are Cohen’s d; significance: *p < 0.05, **p < 0.01; thresholds: 0.2 (small), 0.5 (medium), 0.8 (large).

After the three forest bathing activities, EEG effect sizes showed Group A (combined dynamic-static) showed statistically significant but moderate improvements in positive emotions (Fp1: *d* = 0.645, 95% CI [0.088, 0.959]; F7: *d* = 0.529, 95% CI [0.038, 0.911]; F3: *d* = 0.488, 95% CI [−0.008, 0.971]) and modest reductions in negative emotions (F4: *d* = 0.509, 95% CI [0.010, 0.994]; Fp2: *d* = 0.351, 95% CI [−0.131, 0.822]). Group B (dynamic) and Group C (static) had minimal effects. Group D (control) showed small changes. In summary, Group A tended to show the largest statistical effects among groups, though effect sizes were moderate (Figure 5; Supplementary Table 2 for full results).

#### 3.1.2 Galvanic skin response

Post-forest bathing, GSR effect sizes showed Group A (combined dynamic-static) had the highest (*d* = 0.642, 95% CI [0.085, 0.954]), followed by Group C (static) with *d* = 0.477, 95% CI [−0.021, 0.974]. Group B (dynamic) had the lowest (*d* = 0.089, 95% CI [−0.409, 0.587]). Overall, Group A demonstrated statistically significant changes, although the clinical relevance may be limited due to variability. Yet the pattern suggests that combined activities may elicit stronger autonomic engagement.

#### 3.1.3 Heart rate

Heart rate changes revealed Group B (dynamic) showed statistically significant but small improvements (*d* = 0.136, 95% CI [−0.330, 0.599]), followed by Group C (static) with *d* = 0.128, 95% CI [−0.338, 0.590]. Group A (combined dynamic-static) showed minimal change (*d* = 0.047, 95% CI [−0.416, 0.509]). Although effect sizes were small, the consistency across groups suggests reliable physiological engagement, supporting the robustness of these findings.

#### 3.1.4 Blood pressure

Forest bathing activities produced statistically significant changes in blood pressure, though effect sizes were small to moderate. Group A (combined dynamic-static) had effect sizes of 0.366 (95% CI [−0.117, 0.838]) for systolic and 0.200 (95%CI [−0.270, 0.664]) for diastolic pressure, with modest regulatory effects particularly on systolic pressure. Group B (dynamic) showed a low effect on systolic (*d* = 0.073, 95% CI [−0.391, 0.535]) and a medium effect on diastolic pressure (*d* = 0.292, 95% CI [−0.184, 0.760]). Group C (static) had effect sizes of 0.351 (95% CI [−0.130, 0.823]) for systolic and 0.211 (95% CI [−0.260, 0.675]) for diastolic pressure, with modest effects particularly on systolic pressure. Groups A and C showed moderate regulatory effects, while Group B had the most impact on diastolic pressure. Even modest blood pressure reductions are consistent with prior evidence and may contribute to meaningful long-term health benefits.

### 3.2 Comparison of psychological effects of different activity types on middle-aged and older adult groups

#### 3.2.1 BPOMS mood questionnaire

In the BPOMS mood questionnaire, Group A (combined dynamic-static) showed statistically significant reductions in negative emotions and TMD, with effect sizes of 1.669 (95% CI [0.935, 2.381]) for tension-anxiety and 1.646 (95% CI [1.006, 2.272]) for TMD, and moderate increases in vitality. Group B (dynamic) reduced negative emotions and increased vitality with moderate-to-large effect sizes (0.758, 95% CI [0.223, 1.276]) for tension-anxiety and 1.120,95% CI [0.516, 1.704] for TMD). Group C (static) showed the largest reductions in negative emotions, increased vitality (*d* = 2.227, 95%CI [−3.091, −1.343]), and reduced fatigue (*d* = 2.411, 95% CI [1.475, 3.328]), and large effect sizes for tension-anxiety (*d* = 2.012, 95% CI [1.188, 2.817]). Group D (control) showed only small changes (*d* = 0.517, 95% CI [0.018, 1.004] for tension-anxiety and *d* = 0.495, 95% CI [−0.002, 0.979] for TMD) (Supplementary Table 3).

#### 3.2.2 PRS perceived restorativeness scale

The study revealed that the dynamic group (Group B, *d* = 2.590, 95% CI [−3.559, −1.602]) had the highest effect sizes and perceived recovery compared to the combined dynamic-static (Group A, *d* = 0.662, 95% CI [0.142, 1.167]), static (Group C, *d* = 0.948, 95% CI [−1.499, −0.379]), and control (Group D, *d* = 0.5, 95% CI [0.002, 0.984]) groups, indicating statistically significant but varied impacts of different activity types on psychological states. However, PRS captures subjective perceptions of environmental restorativeness, which is a distinct construct from emotional states and physiological indicators; therefore, its peak in the dynamic group does not contradict the broader improvements observed in the combined group. These results suggest that activity type meaningfully shapes perceived psychological restoration, even when effect sizes differ in magnitude.

### 3.3 The impact of different activity types on gender differences in physiological indicators

#### 3.3.1 EEG

**Figure 6:**
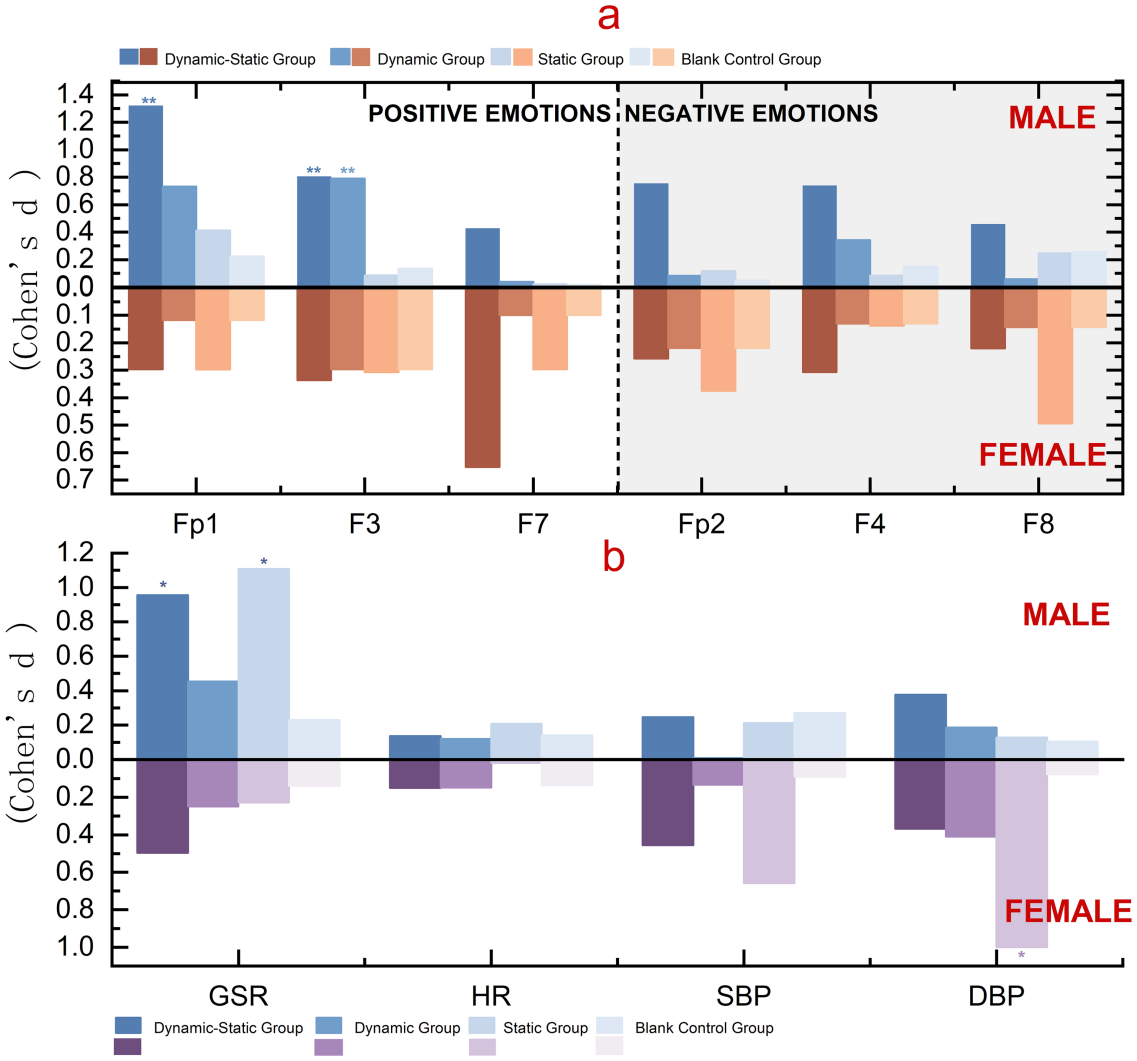
Gender differences in physiological indicators. **(a)** EEG effect sizes at Fp1, F3, F7 (positive-emotion) and Fp2, F4, F8 (negative-emotion), shown separately for males (above zero) and females (below zero). **(b)** Effect sizes for GSR, HR, SBP, and DBP by gender. Bars represent the four groups. Values are Cohen’s d; significance: *p < 0.05, **p < 0.01.

EEG effect sizes revealed gender differences in emotional responses to forest activities. Combined dynamic-static activities (Group A) had statistically significant effects for both genders, with males showing larger positive emotion effects (Fp1: *d* = 1.319, 95% CI [0.388, 2.210]) and greater reductions in negative emotions (Fp2: *d* = 0.754, 95% CI [0.006, 1.457]). Single dynamic activities (Group B) showed the most impact on males (Fp1: *d* = 0.731, 95% CI [−0.028, 1.456]). Static activities (Group C) were more effective for females, increasing positive emotions (Fp1: *d* = 0.297, 95% CI [−0.381, 0.956]) and reducing negative emotions (Fp2: *d* = 0.375, 95% CI [−0.300, 1.028]). Group D (control) showed minimal changes (Figure 6; Supplementary Table 4). Given subgroup sizes (*n* = 9), these gender-specific findings should be considered exploratory.

#### 3.3.2 GSR

GSR effect sizes showed males had higher responses across all groups, with static activities (Group C) showing the highest for males (*d* = 1.107, 95% CI [0.336, 1.828]) and combined dynamic-static activities (Group A) the highest for females (*d* = 0.495, 95% CI [−0.269, 1.199]). All activity groups outperformed the control group. However, subgroup results are based on small samples and require cautious interpretation.

#### 3.3.3 HR

Heart rate effect sizes suggested gender-specific patterns, although the magnitudes were small. Males tended to show greater recovery in Group C (static, *d* = 0.208, 95% CI [−0.949, 0.590]), while females showed relatively higher responses in Groups A (combined dynamic-static, *d* = 0.150, 95% CI [−0.613, 0.874]) and B (dynamic, *d* = 0.148, 95% CI [−0.615, 0.872]). While all confidence intervals crossed zero, the consistent directional trends indicate potential gender-related differences in autonomic regulation. These preliminary findings warrant further investigation with larger samples to clarify the robustness of gender effects in HR responses.

#### 3.3.4 BP

Forest bathing activities suggested potential gender differences in blood pressure responses. Females exhibited relatively higher effects, particularly in static activities (systolic: *d* = 0.658, 95% CI [−0.118, 1.375]; diastolic: *d* = 0.999, 95% CI [0.235, 1.758]). Males showed modest diastolic pressure changes in combined dynamic-static (*d* = 0.376, 95% CI [−1.128, 0.402]) and static activities (*d* = 0.127, 95% CI [−0.889, 0.645]). Although these subgroup findings are based on small samples and should be interpreted with caution, the consistent patterns suggest that females may benefit more from static activities, while males show relatively greater tendencies toward diastolic regulation.

### 3.4 The impact of different activity types on gender differences in psychological indicators

#### 3.4.1 Emotional indicators

**Figure 7:**
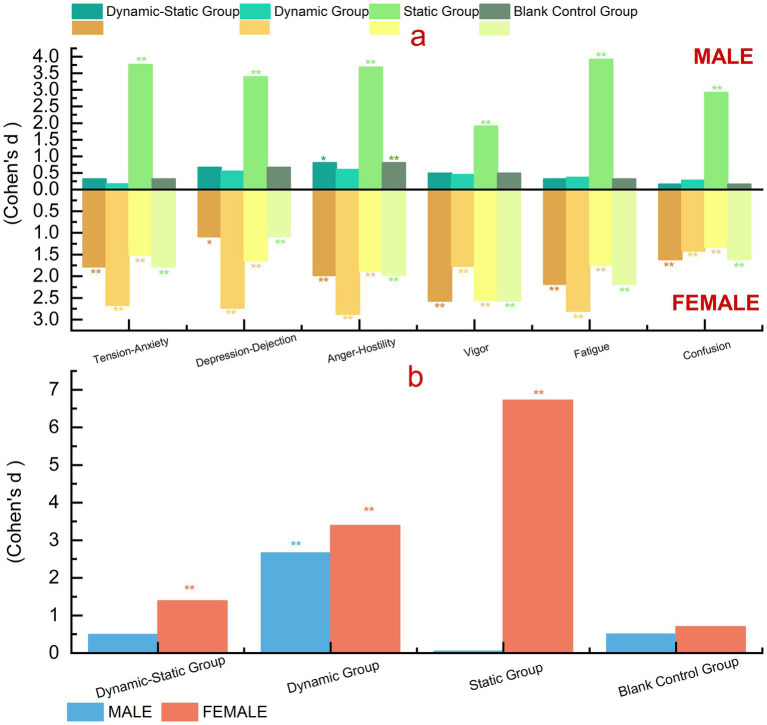
Gender differences in psychological indicators. **(a)** BPOMS effect sizes across subscales (tension–anxiety, depression–dejection, anger–hostility, vigor, fatigue, confusion), shown separately for males (above zero) and females (below zero) in four groups (Dynamic–Static, Dynamic, Static, Blank Control). **(b)** PRS effect sizes across groups, shown separately for males (blue) and females (orange). Values are Cohen’s d; significance: *p < 0.05, **p < 0.01.

Forest bathing activities showed statistically significant effects on psychological indicators, with gender differences observed. Combined dynamic-static and dynamic activities were associated with improvements in females’ psychological health, with effect sizes up to 2.874 (95% CI [1.86, 3.86]) for anger-hostility and 2.805 (95% CI [1.82, 3.79]) for fatigue. Static activities showed marked reductions in males’ negative emotions, with effect sizes of 3.766 (95% CI [2.36, 5.13]) for tension-anxiety and 3.689 (95% CI [2.30, 5.06]) for anger-hostility. Control group results supported these benefits (Figure 7; Supplementary Table 5).

#### 3.4.2 Perceived restorativeness indicators

We evaluated gender differences in perceived restorativeness (PRS) for different forest bathing activities, showing significant differences. Females consistently reported higher PRS across all groups, with the static group showing the largest effect size (*d* = 6.723, 95% CI [5.10, 8.35] for females vs. *d* = 0.048, 95% CI [−0.72, 0.82] for males), indicating substantial perceived psychological benefits for females. These findings, while based on small subgroups, reveal a consistent trend that merits confirmation in larger samples.

## 4 Discussion

### 4.1 Mechanisms of the impact of forest bathing environments on human health

Forest bathing improves both physiological and psychological health by enhancing immune function and reducing disease incidence through high humidity, negative ions, and plant-released (Wroblewska and Jeong, 2021; Yang et al., 2024). Dynamic and static forest activities, such as walking and meditation, optimize health by regulating the autonomic nervous system, increasing parasympathetic activity, and decreasing sympathetic activity. Forest walking boosts high-frequency heart rate variability and reduces the low-frequency/high-frequency ratio, lowering heart rate and stress hormones, thus benefiting overall health (Li Q., 2023). However, these mechanisms vary due to individual differences, which warrants further investigation.

In this study, we assessed these mechanisms using a set of validated physiological and psychological indicators. Our findings indicate that forest bathing enhances parasympathetic activity, reduces sympathetic activity, decreases negative emotions, increases positive emotions, and improves environmental perception, reinforcing its holistic health benefits (Chen et al., 2024; Donelli et al., 2023). However, individual differences in physiological responses, particularly in older adults, highlight the need for further exploration of age-related variations in autonomic nervous system sensitivity. Future studies could also incorporate additional biomarkers, such as cortisol levels or heart rate variability (HRV) over longer durations, to provide a more comprehensive understanding of forest bathing’s long-term effects.

### 4.2 Differences in health effects of forest bathing under different modes

Forest bathing benefits health through environmental and intervention effects. This study tested dynamic, static, and combined activities with varying physical exertion to assess their impact on physical and mental health. Results showed all three types had positive effects, with some differences among them.

#### 4.2.1 Combined dynamic-static group (Group A)

The combined dynamic-static group showed significant improvements in emotional outcomes and blood pressure regulation, with moderate effects on GSR. However, heart rate changes were minimal, and perceived restorativeness (PRS) was lower than in the static group. This likely reflects construct differences, as PRS emphasizes subjective environmental perceptions, whereas the combined group produced broader and more balanced benefits across physiological and psychological indicators. These outcomes may also be influenced by the shorter exposure to each activity compared with single-mode groups, transitional rest periods that rebalanced physiological responses (Song et al., 2017), and the additional cognitive load of switching activities for older adults. Taken together, the combined mode does not simply yield additive effects but instead provides a more integrated outcome, reflecting the complex nature of real-world forest therapy practices.

#### 4.2.2 Dynamic group (Group B)

The study found that moderate dynamic activities like forest walking reduce stress hormones, promote relaxation, and balance the autonomic nervous system. They increase high-frequency heart rate variability, lower the low-frequency to high-frequency ratio, and reduce systolic and diastolic blood pressure (Wen et al., 2023).

In our study, dynamic activities notably improved heart rate and diastolic blood pressure, while EEG and GSR effects were minimal, possibly due to short exposure and lower sweating rates in older adults. The BPOMS results indicated reduced negative emotions and increased vitality, though overall benefits were greater in the combined group. PRS scores were highest for the dynamic group, reflecting that walking more strongly evokes immediate perceptions of restorativeness, even if comprehensive improvements were more evident in the combined mode.

#### 4.2.3 Static group (Group C)

Research shows that static activities like forest meditation can lower blood pressure, heart rate, and cortisol levels, reduce stress, anxiety, depression, and negative emotions, and increase calmness and happiness. These activities enhance immune function, boost natural killer cell activity, and improve attention and cognitive flexibility (Wen et al., 2019; Park et al., 2022; Stier-Jarmer et al., 2021; Twohig-Bennett and Jones, 2018).

Static activities offer varied benefits, with effects differing by individual, particularly in middle-aged and older females. Our study on single static activities found low EEG effect sizes, moderate GSR effects, and insignificant heart rate reductions, likely due to participants’ age. Static activities significantly regulated systolic blood pressure and reduced negative emotions and fatigue, improving vitality. Perceived restorativeness was high but lower than dynamic activities, possibly due to older adults’ preference for shorter-duration static activities.

### 4.3 Gender differences in physiological and psychological responses to forest bathing

Forest bathing’s restorative benefits may vary by gender in exploratory ways, with males tolerating higher temperatures and females preferring lower ones. Variations in light and temperature, especially during increased activity, appear to affect genders differently (Erfanian et al., 2019). Males and females have different thermoregulation and heart rate responses during exercise. Females cool down faster and feel colder in cooler environments despite similar core temperature and heart rate changes (Cernych et al., 2017).

Males generally excel in visual–spatial skills like rotating 3D shapes and tracking objects, which may enhance their forest exploration performance (Weisberg et al., 2011). Females are more prone to psychological distress, such as anxiety and depression, and have higher left amygdala activity, linked to stronger emotional memory and richer experiences (Shafiq et al., 2023). Females are more likely to show psychological distress under stress and negative emotions, such as during pandemics, with notable differences in stress and coping strategies compared to males (Roviello et al., 2022).

This study provides exploratory evidence suggesting that males and females respond differently to forest environments due to physiological and psychological variations. Physiologically, males may benefit more from dynamic activities due to their higher thermoregulation efficiency and cardiovascular response (Wen et al., 2023; Park et al., 2010; Song et al., 2016), while females may find greater restorative effects in static activities such as meditation, which are consistent with their stronger emotional processing and stress sensitivity (Ochiai et al., 2015). Psychologically, females, being more prone to anxiety and emotional distress, may require interventions that emphasize emotional regulation, such as guided relaxation or nature-based mindfulness practices. These gender differences may provide useful guidance for designing personalized forest bathing programs. For instance, health interventions could tailor activities based on gender-specific needs: women may benefit more from structured mindfulness-based forest therapy, while men may experience greater relaxation through exploratory and physically engaging activities. Future research should further explore how to optimize forest therapy protocols based on gender-specific physiological and psychological responses, ensuring that both men and women maximize the restorative benefits of nature exposure.

#### 4.3.1 Combined dynamic-static group (Group A)

Long-term or intense forest bathing may reveal trend-level psychological and physiological differences between genders. Males tended to show greater positive emotional response to combined dynamic-static activities, while females appeared to experience a more significant reduction in negative emotions, indicating better acceptance.

GSR results revealed exploratory evidence of higher effects in males due to greater sweat gland density and sweating rates, leading to more pronounced changes. Differences in thermoregulation and skin properties also may affect GSR sensitivity (Erfanian et al., 2019; Bari, 2020; Horvers et al., 2021; Posada-Quintero et al., 2018).

Females had slightly higher heart rates than males, with minimal difference. Males’ heart rates appeared to recover faster post-exercise, likely due to greater cardiac efficiency, while females’ recovery was slower, possibly due to lower cardiac output and smaller heart volume.

In combined dynamic-static activities, females had higher systolic but slightly lower diastolic blood pressure than males, with slower increases during exercise. These findings suggest a trend that forest bathing activities should be tailored to gender-specific characteristics.

The BPOMS questionnaire indicated that combined dynamic-static activities significantly improved positive emotions and reduced negative emotions in females, with greater total mood disturbance (TMD) improvement in females compared to males. Females may be more responsive to emotional regulation, using emotion-focused strategies like seeking support and expressing feelings. The sensory stimuli and social interaction from combined activities seem to help females alleviate negative emotions more effectively (Deng et al., 2016). Males often manage stress alone or via exercise, resulting in less emotional recovery than females. The perceived restorativeness questionnaire also showed that females tended to report significantly higher recovery effects than males (Deng et al., 2016).

#### 4.3.2 Dynamic group (Group B)

Moderate dynamic activities like forest walking release stress hormones, trigger relaxation, reduce sympathetic activity, and enhance parasympathetic activity, lowering heart rate and blood pressure while boosting self-regulation. Dynamic forest bathing increased high-frequency heart rate variability, reduced the LF/HF ratio, and lowered systolic and diastolic blood pressure, and pulse rate (Wen et al., 2023).

In our study, dynamic activities notably impacted heart rate, but systolic blood pressure results differed from previous studies, with diastolic blood pressure showing the greatest improvement. Factors such as the national forest park setting, experiment duration, uncontrolled walking speed, and the older age of participants may explain these differences. EEG data revealed that single dynamic activities had the smallest effect on emotions, likely due to the limited emotional change they induce in older adults. GSR results also indicated the lowest effect size for single dynamic activities, possibly due to lower sweating rates and less sensitive skin responses in the older adult. The BPOMS questionnaire showed that single dynamic activities reduced negative emotions and increased vitality, but less effectively than combined activities. However, single dynamic activities had the highest restorative effects in the perceived restorativeness questionnaire, likely due to their nature.

#### 4.3.3 Static group (Group C)

In single static activities (e.g., forest meditation), males and females show trend-level differences in physiological and psychological responses. EEG data revealed that static activities significantly improved positive emotions in females but had a smaller effect on males. This may be because females use static activities for emotional regulation, while males prefer dynamic activities for stress relief (Kivikangas et al., 2014). GSR responses showed that males had a higher effect size during static activities, indicating they sweated more, which could be related to their higher basal metabolic rate and sympathetic nervous system response (Bari, 2020; Posada-Quintero et al., 2018).

For heart rate recovery, males showed a higher effect size than females, suggesting static activities might have been more effective for them. This may be due to better cardiac function in males, enhancing their recovery from static activities (Bari, 2020; Posada-Quintero et al., 2018). Static activities significantly regulated blood pressure in females, particularly diastolic pressure, likely due to their ability to relax and lower blood pressure more effectively (Posada-Quintero et al., 2018).

The BPOMS questionnaire showed that while males had a higher effect size for reducing negative emotions, they had lower vitality compared to females. Single static activities improved overall emotional health more for females, likely due to their greater sensitivity to emotional regulation and use of emotion-focused coping strategies. The perceived restorativeness questionnaire revealed that females tended to report significantly higher restorative effect than males, indicating better emotional recovery and regulation during static activities.

These differences are consistent with the idea that fundamental physiological and psychological variations between genders shape forest therapy responses. Males seem to exhibit stronger physiological responses in static activities, while females may excel in emotional regulation and recovery (Deng et al., 2016; Kivikangas et al., 2014). Therefore, forest bathing activities should consider these gender differences as potential factors to maximize health benefits.

Although several indicators showed statistically significant changes, some effect sizes (e.g., heart rate and EEG) were modest. This distinction highlights the difference between statistical and clinical significance. However, even small but consistent changes in physiological and psychological indicators may still be meaningful for middle-aged and older adult populations, where incremental improvements accumulate to long-term health benefits. Importantly, the overall trends across multiple measures were coherent and aligned with previous findings, supporting the exploratory robustness our results. Future studies with larger sample sizes and longer follow-up periods are needed to further establish the clinical relevance and generalizability of these findings.

### 4.4 Uniqueness and contribution of the study

Unlike previous studies, our research examines the physiological and psychological effects of dynamic, static, and combined forest bathing activities across genders, offering gender-specific recommendations. Key contributions include:

1) Real-world Setting: The study was conducted in a national forest park — specifically, the Panda Valley in Foping County, Shaanxi Province, located within the transition zone of the Qinling Mountains. This region is part of a typical ecological area within China’s national forest park system, featuring high biodiversity and well-preserved forest ecosystems. The natural setting ensures ecological validity and enhances the authenticity of the findings.
2) Specific Population: Focuses on middle-aged and older adult individuals, a key demographic in public health that is particularly sensitive to environmental and psychological interventions. The study contributes to developing targeted health strategies for aging populations.
3) Precision in Physiological Measurements: Utilized the SAGA 64 + EEG device with wet electrodes for high-fidelity physiological data, including EEG, heart rate (HR), galvanic skin response (GSR), and blood pressure (SBP, DBP), enhancing reliability in natural field conditions.
4) Combined Dynamic-Static Design: The study innovatively integrates static (e.g., sitting meditation) and dynamic (e.g., walking) activities, as well as their combination, to evaluate their differential impact. This approach fills a gap in current research, which often isolates single activity types.
5) Gender Differences Analysis: Detailed analysis of gender-specific physiological and psychological responses provides valuable insights into the design of personalized forest therapy programs, addressing the differential health needs of males and females.
6) Integration with National Forest Park Health Strategies: By grounding the study in a real national forest park, this research contributes to the growing body of evidence that positions national forest parks as important therapeutic landscapes. The findings support the integration of nature-based therapy into broader public health and environmental management frameworks.
7) Policy and Practical Relevance: The study provides scientific and data-driven recommendations for designing forest-based wellness programs, helping to guide the development of health services within national forest parks. It supports the dual goals of ecological preservation and human well-being, aligning with China’s strategy to expand the health value of protected natural areas.

### 4.5 Limitations

This study was conducted in a national forest park, which enhanced ecological validity, but several limitations should be noted. First, we mainly assessed short-term effects and lacked long-term follow-up. Future studies should include longitudinal and multi-center designs to verify the sustainability of forest bathing benefits.

Second, certain environmental factors (e.g., plant-derived volatile compounds, weather, and soundscapes) could not be fully controlled. Although we standardized intervention areas, walking routes, and times, and monitored air quality and noise levels, residual confounding cannot be ruled out.

Third, aspects of the intervention design may have introduced bias. The combined activity group had a longer total duration than single-mode groups, raising the possibility of time as a confounder. The target walking speed in the dynamic group (≈6 km/h) may have posed a greater physical challenge for some older participants, yet functional capacity was not formally stratified. In the static group, expert-evaluated audio was delivered via headphones to ensure standardization, but this excluded natural forest sounds. Future research should consider equal-duration comparisons, stratification or individualized walking speeds, and incorporating natural soundscapes.

Fourth, individual behaviors such as walking pace, verbal interactions, and meditation focus could not be fully standardized. While we minimized variability through standardized routes, demonstrations, and guided audio, individual differences may still have influenced outcomes. Importantly, such variation also reflects the authentic experiential nature of forest bathing.

Fifth, the study relied primarily on self-reported measures, which may be subject to bias. More objective physiological and behavioral assessments are needed in future work.

Sixth, sample size was limited to 18 per group, consistent with previous studies, but gender subgroups included only nine participants, resulting in reduced statistical power. Subgroup results should therefore be interpreted as exploratory. Larger, multi-center studies with systematic stratified analyses are warranted to enhance robustness and external validity.

Finally, findings were based on the Panda Valley forest park, a specific type of forest environment. Effects of dynamic, static, and combined activities may differ in other forest types (e.g., coniferous, broadleaf, mixed) or populations (e.g., younger adults, patients with chronic conditions). Cross-site and cross-population validation is needed to strengthen generalizability.

Despite these limitations, the study provides valuable empirical evidence on the physiological and psychological effects of different activity types and gender differences in forest bathing, offering a basis for future large-scale and diversified research.

## 5 Conclusion

This study demonstrates that forest bathing activities, including dynamic, static, and combined dynamic-static activities, have significant restorative effects on the psychological and physiological health of older adult populations, with notable differences depending on the activity type. Combined dynamic-static activities provide the most comprehensive restorative benefits, while dynamic and static activities excel in specific health indicators.

The study also reveals that gender influences the restorative effects. Males show more pronounced improvements in physiological indicators, whereas females generally outperform males in psychological restoration. These findings suggest that the design and promotion of forest bathing activities should consider gender differences to offer more personalized and effective restorative programs.

Importantly, this study was conducted in a national forest park with rich biodiversity and ecological integrity, reinforcing the role of protected natural environments as therapeutic landscapes. The findings provide empirical evidence that supports the integration of nature-based therapies into national forest park planning and public health strategies, aligning with the broader goal of enhancing the health value of protected areas.

Future forest bathing service designs should incorporate both individual and combined activity options to meet diverse participant needs. Tailoring forest bathing programs based on gender and individual health conditions will likely enhance participant satisfaction and health outcomes. Initial assessments of participants’ preferences and needs can guide the development of effective wellness programs, ensuring that activity design maximizes physical and mental health benefits. Additionally, site selection should be diverse, including open walking paths and quiet meditation areas, with rich natural elements to enhance sensory restoration experiences.

## Data Availability

Most of the data supporting the findings of this study are included in the article. A subset of the data concerning gender-related analyses is available from the corresponding author upon reasonable request, as these data are being used for further ongoing research.
