# Supplementary material for: The differential effects of dynamic, static, and combined activities in forest bathing on health outcomes by gender in older adults: evidence from a national forest park trial
Source: Front Psychol. 2025 Oct 22;16:1648144. doi: 10.3389/fpsyg.2025.1648144 (PMC12593491; doi:10.3389/fpsyg.2025.1648144)
Supplement: Supplementary file 2 [file Supplementary_file_1.zip › Revised_Supplementary_Tables_v2/Table A.1.docx]

**Table A.1: Initial baseline**

|  |  | **Group mean ± standard deviation** | | | **F** | ***p*** |
| --- | --- | --- | --- | --- | --- | --- |
|  |  | **A(n=18)** | **B(n=18)** | **C(n=18)** |  |  |
| **Psychological indicators** | | | | | | |
| **PRS** | pre | 2.90±1.03 | 2.68±0.09 | 2.68±0.17 | 0.783 | 0.462 |
| **BPOMS** | pre | 58.50±16.43 | 63.94± 2.86 | 63.22±6.85 | 1.45 | 0.2433 |
| **Physiological indicators** | | | | | | |
| **GRS** | pre-test | 816.98±254.51 | 687.89±355.30 | 826.76±258.50 | 1.258 | 0.293 |
| **HR** | pre-test | 83.61±13.57 | 78.17±12.28 | 77.22±14.54 | 1.175 | 0.317 |
| **BP(DBP)** | pre-test | 75.61±9.39 | 78.22±13.37 | 80.61±10.04 | 0.918 | 0.406 |
| **BP(SBP)** | pre-test | 132.33±22.91 | 122.33±32.53 | 131.33±20.28 | 0.821 | 0.446 |
| **Fp1** | pre-test | 25.18±19.02 | 25.18±19.02 | 15.39±22.63 | 1.395 | 0.257 |
| **Fp2** | pre-test | 24.46±18.59 | 24.46±18.59 | 16.05±22.27 | 1.073 | 0.35 |
| **F7** | pre-test | 22.84±17.44 | 22.84±17.44 | 10.06±25.95 | 2.294 | 0.111 |
| **F3** | pre-test | 25.69±20.63 | 25.69±20.63 | 12.75±29.94 | 1.726 | 0.188 |
| **F4** | pre-test | 24.43±15.89 | 24.43±15.89 | 17.67±20.92 | 0.873 | 0.424 |
| **F8** | pre-test | 23.95±18.17 | 23.95±18.17 | 19.74±22.68 | 0.271 | 0.764 |

* *p*<0.05 ** *p*<0.01
