# Supplementary material for: The differential effects of dynamic, static, and combined activities in forest bathing on health outcomes by gender in older adults: evidence from a national forest park trial
Source: Front Psychol. 2025 Oct 22;16:1648144. doi: 10.3389/fpsyg.2025.1648144 (PMC12593491; doi:10.3389/fpsyg.2025.1648144)
Supplement: Supplementary file 2 [file Supplementary_file_1.zip › Revised_Supplementary_Tables_v2/Table A.3 .docx]

**Table A.3. Effect Sizes (Cohen’s d) and 95% Confidence Intervals for Psychological Indicators (BPOMS Subscales and PRS) Across Groups**

|  |  | **A（Dynamic-Static Group）** | | **B（Dynamic Group）** | | **C（Static Group）** | | **D （Blank Control Group）** | |
| --- | --- | --- | --- | --- | --- | --- | --- | --- | --- |
|  |  | **Cohen's d** | **95% CI** | **Cohen's d** | **95% CI** | **Cohen's d** | **95% CI** | **Cohen's d** | **95% CI** |
| **BPOMS** | | | | | | | | | |
| TA | | 1.669 | [0.935, 2.381] | 0.758 | [0.223, 1.276] | 2.012 | [1.188, 2.817] | 0.517 | [0.018, 1.004] |
| DD | | 1.241 | [0.618, 1.864] | 1.103 | [0.511, 1.695] | 0.870 | [0.323, 1.417] | 0.196 | [−0.271, 0.663] |
| AH | | 1.55 | [0.854, 2.246] | 1.121 | [0.525, 1.717] | 2.498 | [1.540, 3.456] | 0.097 | [0.272, 1.346] |
| V | | 0.009 | [−0.454, 0.463] | 0.932 | [0.374, 1.490] | 2.227 | [−3.091, −1.343] | 0.425 | [−0.059, 0.909] |
| F | | 0.668 | [0.154, 1.182] | 0.995 | [0.425, 1.565] | 2.411 | [1.475, 3.328] | 0.458 | [−0.030, 0.946] |
| C | | 1.12 | [0.524, 1.716] | 0.618 | [0.113, 1.123] | 0.872 | [0.324, 1.420] | 0.685 | [0.169, 1.201] |
| TMD | | 1.646 | [1.006, 2.272 | 1.120 | [0.516, 1.704] | 0.857 | [0.312, 1.402] | 0.495 | [−0.002, 0.979] |
| **PRS** | | | | | | | | | |
|  | | 0.662 | [0.142, 1.167] | 2.590 | [−3.559, −1.602] | 0.948 | [−1.499, −0.379] | 0.50 | [0.002, 0.984] |

Note: BPOMS = Brief Profile of Mood States (subscales: Tension-Anxiety, Depression-Dejection, Anger-Hostility, Vigor, Fatigue, Confusion; TMD = Total Mood Disturbance). PRS = Perceived Restorativeness Scale. Cohen’s d values are reported with 95% confidence intervals. Positive values indicate improvement (e.g., increased vigor, decreased negative emotions), while negative values reflect symptom reduction due to coding direction. A = Dynamic-Static Group; B = Dynamic Group; C = Static Group; D = Blank Control Group.
