# Supplementary material for: The differential effects of dynamic, static, and combined activities in forest bathing on health outcomes by gender in older adults: evidence from a national forest park trial
Source: Front Psychol. 2025 Oct 22;16:1648144. doi: 10.3389/fpsyg.2025.1648144 (PMC12593491; doi:10.3389/fpsyg.2025.1648144)
Supplement: Supplementary file 2 [file Supplementary_file_1.zip › Revised_Supplementary_Tables_v2/Table A.4.docx]

**Table A.4: Gender Differences in Physiological Indicators: Effect Sizes (Cohen’s d) and 95% Confidence Intervals**

|  |  |  | **A（Dynamic-Static Group）** | | **B（Dynamic Group）** | | **C（Static Group）** | | **D （Blank Control Group）** | |
| --- | --- | --- | --- | --- | --- | --- | --- | --- | --- | --- |
|  |  |  | **Cohen's d** | **95% CI** | **Cohen's d** | **95% CI** | **Cohen's d** | **95% CI** | **Cohen's d** | **95% CI** |
| **EEG** | | | | | | | | | | |
| Fp1 | | Male | 1.319 | [0.388, 2.210] | 0.731 | [−0.028, 1.456 | 0.412 | [−0.283, 1.083] | 0.224 | [−0.879, 0.445] |
|  |  | Female | 0.296 | [−0.381,0.95] | 0.117 | [−0.769, 0.543] | 0.297 | [−0.381, 0.956 | 0.117 | [−0.769, 0.543] |
| F7 | | Male | 0.426 | [−0.373, 1.212] | 0.038 | [−0.682, 0.757] | 0.019 | [−0.702, 0.739] | 0.013 | [−0.706, 0.732] |
|  |  | Female | 0.652 | [−0.157, 1.452] | 0.099 | [−0.630, 0.820] | 0.296 | [−0.382, 0.955] | 0.099 | [−0.620, 0.819] |
| F3 | | Male | 0.803 | [0.018, 1.649] | 0.789 | [0.014, 1.555] | 0.085 | [−0.637, 0.805] | 0.133 | [−0.586, 0.853] |
|  |  | Female | 0.336 | [−0.451, 1.124] | 0.296 | [−0.432, 1.018] | 0.307 | [−0.371, 0.967] | 0.296 | [−0.423, 1.016] |
| Fp2 | | Male | 0.754 | [0.006, 1.457] | 0.081 | [−0.646, 0.813] | 0.116 | [−0.606, 0.838] | 0.049 | [−0.670, 0.768] |
|  |  | Female | 0.257 | [−0.532, 1.043] | 0.219 | [−0.510, 0.943] | 0.375 | [−0.300, 1.028] | 0.219 | [−0.500, 0.939] |
| F4 | | Male | 0.738 | [−0.058, 1.525] | 0.341 | [−0.389, 1.067] | 0.084 | [−0.639, 0.806] | 0.149 | [−0.570, 0.869] |
|  |  | Female | 0.307 | [−0.481, 1.095] | 0.13 | [−0.597, 0.852] | 0.138 | [−0.584, 0.860] | 0.13 | [−0.589, 0.850] |
| F8 | | Male | 0.457 | [−0.340, 1.236] | 0.057 | [−0.670, 0.782] | 0.246 | [−0.479, 0.968] | 0.257 | [−0.463, 0.977] |
|  |  | Female | 0.22 | [−0.576, 1.010] | 0.143 | [−0.585, 0.866] | 0.494 | [−0.236, 1.217] | 0.143 | [−0.577, 0.862] |
| **GRS** | | | | | | | | | | |
| GRS | | Male | 0.955 | [0.122, 1.770] | 0.453 | [−0.282, 1.179] | 1.107 | [0.336, 1.828] | 0.23 | [−0.489, 0.949] |
|  |  | Female | 0.495 | [−0.269, 1.199] | 0.248 | [−0.480, 0.974] | 0.228 | [−0.494, 0.949] | -0.140 | [−0.859, 0.579] |
| **HR** | | | | | | | | | | |
| HR | | Male | 0.135 | [−0.653, 0.918] | 0.119 | [−0.607, 0.844] | 0.208 | [−0.949, 0.590] | 0.14 | [−0.579, 0.859] |
|  |  | Female | 0.150 | [−0.613, 0.874] | 0.148 | [−0.615, 0.872] | 0.017 | [−0.706, 0.739] | 0.136 | [−0.583, 0.855] |
| **BP** | | | | | | | | | | |
| SBP | | Male | 0.246 | [−0.540, 1.028] | 0.008 | [−0.718, 0.735] | 0.212 | [−0.513, 0.936] | 0.270 | [−0.450, 0.989] |
|  |  | Female | 0.455 | [−0.337, 1.240] | 0.133 | [−0.594, 0.856] | 0.658 | [−0.118, 1.375] | 0.090 | [−0.629, 0.809] |
| DBP | | Male | 0.376 | [−1.128, 0.402] | 0.185 | [−0.542, 0.911] | 0.127 | [−0.889, 0.645] | 0.103 | [−0.616, 0.822] |
|  |  | Female | 0.368 | [−0.424, 1.149] | 0.409 | [−0.323, 1.132] | 0.999 | [0.235, 1.758] | 0.077 | [−0.616, 0.822] |

Notes: Cohen’s d values are reported separately for males and females across four groups: Group A (Dynamic–Static), Group B (Dynamic), Group C (Static), and Group D (Blank Control Group). Positive values indicate greater improvements in physiological responses. 95% CIs are based on subgroup sample sizes (n = 9 per gender).
