# Supplementary material for: The differential effects of dynamic, static, and combined activities in forest bathing on health outcomes by gender in older adults: evidence from a national forest park trial
Source: Front Psychol. 2025 Oct 22;16:1648144. doi: 10.3389/fpsyg.2025.1648144 (PMC12593491; doi:10.3389/fpsyg.2025.1648144)
Supplement: Supplementary file 2 [file Supplementary_file_1.zip › Revised_Supplementary_Tables_v2/Table A.5.docx]

|  | | **A（Dynamic-Static Group）** | | **B（Dynamic Group** | | **C（Static Group）** | | **D （Blank Control Group）** | |
| --- | --- | --- | --- | --- | --- | --- | --- | --- | --- |
|  |  | **Cohen's d** | **95% CI** | **Cohen's d** | **95% CI** | **Cohen's d** | **95% CI** | **Cohen's d** | **95% CI** |
| **BPOMS** | | | | | | | | | |
| TA | Male | 0.319 | [−0.45, 1.05] | 0.168 | [−0.59, 0.93] | 3.766 | [2.36, 5.13] | 0.319 | [−0.45, 1.05] |
|  | Female | 1.783 | [0.81, 2.70] | 2.667 | [1.66, 3.65] | 1.514 | [0.57, 2.43] | 1.783 | [0.81, 2.70] |
| DD | Male | 0.667 | [−0.15, 1.46] | 0.55 | [−0.24, 1.30] | 3.395 | [2.05, 4.75] | 0.667 | [−0.15, 1.46] |
|  | Female | 1.09 | [0.23, 1.89] | 2.733 | [1.72, 3.73] | 1.65 | [0.68, 2.59] | 1.09 | [0.23, 1.89] |
| AH | Male | 0.809 | [0.01, 1.57] | 0.601 | [−0.18, 1.36] | 3.689 | [2.30, 5.06] | 0.809 | [0.01, 1.57] |
|  | Female | 1.979 | [0.96, 2.95] | 2.874 | [1.86, 3.86] | 1.878 | [0.85, 2.89] | 1.979 | [0.96, 2.95] |
| V | Male | 0.488 | [−0.29, 1.23] | 0.444 | [−0.32, 1.20] | 1.91 | [0.90, 2.89] | 0.488 | [−0.29, 1.23] |
|  | Female | 2.573 | [1.47, 3.64] | 1.768 | [0.84, 2.70] | 2.556 | [1.47, 3.65] | 2.573 | [1.47, 3.64] |
| F | Male | 0.319 | [−0.45, 1.05] | 0.369 | [−0.40, 1.14] | 3.92 | [2.50, 5.29] | 0.319 | [−0.45, 1.05] |
|  | Female | 2.182 | [1.19, 3.12] | 2.805 | [1.82, 3.79] | 1.75 | [0.74, 2.73] | 2.182 | [1.19, 3.12] |
| C | Male | 0.162 | [−0.60, 0.91] | 0.277 | [−0.49, 1.03] | 2.922 | [1.72, 4.12] | 0.162 | [−0.60, 0.91] |
|  | Female | 1.619 | [0.69, 2.50] | 1.419 | [0.49, 2.31] | 1.325 | [0.38, 2.23] | 1.619 | [0.69, 2.50] |
| TMD | Male | 1.094 | [0.21, 1.90] | 1.167 | [0.28, 1.96] | 5.945 | [4.15, 7.68] | 1.094 | [0.21, 1.90] |
|  | Female | 2.978 | [1.93, 4.01] | 5.19 | [3.75, 6.63] | 0.810 | [−0.05, 1.63] | 2.978 | [1.93, 4.01] |
| **PRS** | | | | | | | | | |
|  | Male | 0.494 | [−0.28, 1.23] | 0.500 | [−0.27, 1.27] | 0.048 | [−0.72, 0.82] | 0.048 | [−0.27, 1.27] |
|  | Female | 1.386 | [0.46, 2.27] | 3.393 | [2.27, 4.49] | 6.723 | [5.10, 8.35] | 6.723 | [0.46, 2.27] |

**Table A.5: Gender Differences in Psychological Indicators (BPOMS Subscales and PRS): Effect Sizes (Cohen’s d) and 95% Confidence Intervals**

Note. TA = Tension-Anxiety; DD = Depression-Dejection; AH = Anger-Hostility; V = Vigor; F = Fatigue; C = Confusion; TMD = Total Mood Disturbance; PRS = Perceived Restorativeness Scale. Values represent effect sizes (Cohen’s d) with 95% confidence intervals across groups. p < 0.05 (*), p < 0.01 (**).
