# Supplementary material for: The differential effects of dynamic, static, and combined activities in forest bathing on health outcomes by gender in older adults: evidence from a national forest park trial
Source: Front Psychol. 2025 Oct 22;16:1648144. doi: 10.3389/fpsyg.2025.1648144 (PMC12593491; doi:10.3389/fpsyg.2025.1648144)
Supplement: Supplementary file 2 [file Supplementary_file_1.zip › Revised_Supplementary_Tables_v2/Table A.2.docx]

**Table A.2: Effect Sizes (Cohen’s d) and 95% Confidence Intervals for Physiological Indicators Across Groups**

|  | **A（Dynamic-Static Group）** | | **B（Dynamic Group）** | | **C（Static Group）** | | **D （Blank Control Group）** | |
| --- | --- | --- | --- | --- | --- | --- | --- | --- |
| GSR | **Cohen's d** | **95% CI** | **Cohen's d** | **95% CI** | **Cohen's d** | **95% CI** | **Cohen's d** | **95% CI** |
| Fp1 | 0.645 | [0.088, 0.959] | 0.203 | [−0.294, 0.701] | 0.165 | [−0.332, 0.663] | 0.231 | [−0.266, 0.728] |
| F7 | 0.529 | [0.038, 0.911] | 0.015 | [−0.482, 0.513] | 0.149 | [−0.349, 0.646] | 0.087 | [−0.410, 0.584] |
| F3 | 0.488 | [−0.008, 0.971] | 0.106 | [−0.392, 0.603 | 0.057 | [−0.441, 0.554] | 0.190 | [−0.307, 0.687] |
| Fp2 | 0.351 | [−0.131, 0.822] | 0.09 | [−0.588, 0.407] | 0.143 | [−0.640, 0.354] | 0.118 | [−0.379, 0.615] |
| F4 | 0.509 | [0.010, 0.994] | 0.064 | [−0.561, 0.434] | 0.1 | [−0.598, 0.397] | 0.200 | [−0.297, 0.697] |
| F8 | 0.165 | [−0.298, 0.615] | 0.036 | [−0.461, 0.533] | 0.04 | [−0.538, 0.457] | 0.005 | [−0.492, 0.502] |
| GSR | 0.642 | [0.085, 0.954] | 0.089 | [−0.409, 0.587] | 0.477 | [−0.021, 0.974] | 0.241 | [−0.257, 0.738] |
| HR | 0.047 | [−0.416, 0.509] | 0.136 | [−0.330, 0.599] | 0.128 | [−0.338, 0.590] | 0.09 | [−0.370, 0.556] |
| SBP | 0.366 | [−0.117, 0.838] | 0.073 | [−0.391, 0.535] | 0.351 | [−0.130, 0.823] | 0.140 | [−0.325, 0.604] |
| DBP | 0.200 | [−0.270, 0.664] | 0.292 | [−0.184, 0.760] | 0.211 | [−0.260, 0.675] | 0.090 | [−0.374, 0.552] |

Table A.2. Effect sizes (Cohen’s d) and corresponding 95% confidence intervals for EEG electrodes (Fp1–F8), GSR, HR, SBP, and DBP across four activity groups (A = Combined Dynamic-Static, B = Dynamic, C = Static, D = Blank Control). Positive electrodes (Fp1, F3, F7) indicate positive emotions; negative electrodes (Fp2, F4, F8) indicate negative emotions. Cohen’s d thresholds: 0.2 = small, 0.5 = medium, 0.8 = large.
